# Immunotherapy: From Advanced NSCLC to Early Stages, an Evolving Concept

**DOI:** 10.3389/fmed.2020.00090

**Published:** 2020-03-24

**Authors:** Thierry Berghmans, Valérie Durieux, Lizza E. L. Hendriks, Anne-Marie Dingemans

**Affiliations:** ^1^Clinic of Thoracic Oncology, Institut Jules Bordet, Université Libre de Bruxelles, Brussels, Belgium; ^2^Bibliothèque des Sciences de la Santé, Université Libre de Bruxelles, Brussels, Belgium; ^3^Department of Pulmonary Diseases (GROW), School for Oncology and Developmental Biology, Maastricht University Medical Center+, Maastricht, Netherlands; ^4^Department of Pulmonary Diseases, Erasmus Medical Center, Rotterdam, Netherlands

**Keywords:** non-small cell lung cancer, pembrolizumab, atezolizumab, nivolumab, immunotherapy, checkpoint inhibition

## Abstract

Immunotherapy in lung cancer treatment is a long history paved with failures and some successes. During the last decade, the discovery of checkpoints inhibitors led to major advances in treating advanced and metastatic non-small cell lung cancer (NSCLC). Impressive data from early phase I-II studies were subsequently confirmed in large prospective randomized trials and meta-analyses (High-level of evidence). Three anti- programmed death-1 (PD1) (pembrolizumab, nivolumab) or antiPD-ligand(L)1 (atezolizumab) antibodies showed clinically significant improved survival compared to second-line docetaxel. Then, first-line pembrolizumab monotherapy demonstrated its superiority over platinum-doublet in high PD-L1 NSCLC. The addition of pembrolizumab or atezolizumab to chemotherapy derived the same results regardless of the PD-L1 status. On the opposite, antiCTLA4 (Cytotoxic T-Lymphocyte Associated 4) results are currently disappointing in unselected patients while recent development suggest that the combination of antiPD1 and antiCTLA4 (nivolumab-ipilimumab) positively impact on overall survival. Some secondary analyses also showed that immunotherapy has a positive impact on quality of life and that the clinical improvement can be done at an acceptable incremental cost per QALY. A lot of questions remain unresolved: which is the best treatment duration and is it the same for all patients, how to choose the patients that will have the highest benefit of immunotherapy, how to identify the patients who will have rapid progression, how to improve the current data (new targets, new combinations)…

## Introduction

Lung cancer is the first cause of death by cancer worldwide (1) because most of the patients with non-small cell lung cancer (NSCLC) are diagnosed at advanced stages or are presenting with recurrent disease after initial locoregional treatment. Up to the end of the twentieth century, conventional chemotherapy, mainly platinum-based, was the only therapeutic option for those not eligible for radical intent treatment, with limited efficacy and very few long-term survivors. The discovery of activating oncogenic driver aberrations led to development of very active targeted therapies (2). Unfortunately, these drugs are restricted to relatively rare selected populations, as the most frequent Epidermal Growth Factor Receptor (*EGFR*) sensitive mutations are found in only 10–15% of adenocarcinoma in Caucasian people.

Out of multiple molecular alterations leading to neoplastic transformation, immune escape of the cancer cell is of importance in cancer growth and in the metastatic process (3). Immunotherapy already has a long story with the first empiric treatments administered to cancer patients at the end of the nineteenth century (Cooley toxin) (4). Multiple attempts in modulating the immune response showed disappointing results in lung cancer when using interleukins, interferons or more recently, vaccination strategies (5).

Advances in understanding the immune cycle control (6) led to the discovery of checkpoint inhibitors such as Cytotoxic T-Lymphocyte Associated 4 (CTLA4), Programmed Death-1 (PD1) and Programmed Death-Ligand 1 (PDL1), future targets for immunotherapy (7, 8). Antibodies directed against these targets first demonstrated a major activity in metastatic melanoma (9, 10). Subsequently, other tumors as NSCLC showed sensitivity to these drugs. Different monoclonal antibodies have currently a marketing authorization and others are under investigation. Two antiPD1 are available: nivolumab (BMS-936558, MDX1106, ONO-4538), an IgG4 antibody, and pembrolizumab (MK-3475), also humanized IgG4. AntiPD-L1 antibodies are among others BMS-936559 (IgG4), and IgG1 atezolizumab (MPDL3280A), durvalumab (MEDI4736), and avelumab (MSB001078C). Currently, nivolumab, pembrolizumab, and atezolizumab obtained marketing authorization by the European Medicine agency (EMA) in metastatic NSCLC. The two main antiCTLA4 currently under investigation are ipilimumab and tremelimumab.

Below, we aim to provide an overview on currently available data on immunotherapy efficacy in NSCLC, including a systematic review of published clinical trials. Focus is on antiPD1/PDL1 and antiCTLA4 antibodies alone or in combination with chemotherapy.

## Materials and Methods

Our main objective is presenting a review on the current knowledge about the clinical activity of immunotherapy in NSCLC. In addition to a narrative review, we performed a systematic review restricted to antiPD1/PDL1 and antiCTLA4 antibodies dedicated to clinical trials assessing activity of immunotherapy alone or in combination with chemotherapy. Inclusion selection criteria of the trials are the following: any phase prospective clinical trial; systematic reviews and meta-analyses; inclusion of NSCLC of any stage and any sub-histological type (in case of mixed population, data on activity should be obtained separately for the NSCLC group); study dedicated to antiPD1, antiPDL1, or antiCTLA4, eventually in combination with chemotherapy. Further, data regarding immunotherapy activity must be reported in the manuscript on response rate, progression-free survival (PFS), or overall survival (OS).

The literature search was done in July 2019 using the Ovid Medline system. This research was performed by a scientific librarian (VD) experienced in searching for medical and scientific publications, and by a physician (TB) expert in the treatment of thoracic neoplasms and trained in evidence-based medicine.

Ovid Medline database was searched using the OvidSP interface. The “PICO” (population, intervention, comparator, outcome) model for clinical questions was used to identify the concepts included in the questions (11). The corresponding search criteria of “P” and “I” were translated into MeSH terms, and free-text keywords that were searched for in titles, abstracts and name of substances (Appendix 1 in Supplementary Material). Citations were exported from Medline into a reference manager software to allow the removal of duplicates. All articles retrieved by the librarian were sent to one member of the group (TB). They were first selected for their eligibility based on the abstract content and the language. Only publications accessible to the authors for their language (English, French, Dutch) were deemed eligible. The final selection was made after reading the full publication. Another member of the group (AD) independently confirmed the selection. This search was supplemented by screening the references of the selected articles and other literature known by the experts. There was no selection based on year of publication.

## Results

Nine hundred sixty-six abstracts were retrieved from the literature search. According to inclusion criteria, 62 articles were finally selected. In addition, three major randomized studies known by the authors but only presented in abstract form were retrieved and added to the search (Appendix 2 in Supplementary Material). The articles dedicated to single clinical studies were separated into 4 groups: Phase I-II trials, randomized trials in the salvage setting, randomized trials in first-line setting, and other studies. In addition, 15 selected publications were systematic reviews and meta-analyses while 17 others were not considered as more recent data on the same topic were available.

The results are presented on a logical basis from early studies (phase I-II) to randomized trials in 1st line setting. The data are summarized in Table 1 (phase I-II studies), Table 2 (salvage therapy), Table 3 (first-line), Table 4 (meta-analyses of efficacy), and Table 5 (meta-analyses of compared activity).

**Table 1:** Main characteristics of published phase I-II studies assessing immunotherapy antibodies in NSCLC.

**References**	**Date**	**Phase**	***N* patients**	**Target**	**Drug**	**Setting**	**Schedule/dose**	**RR**	**mPFS**	**MST**
Brahmer et al. (12)	2010	I	39 (6 NSCLC)	PD1	Nivolumab (MDX-1106)	>1 line	0.3–10 mg/kg	7.7% (whole group)	–	–
Topalian et al. (13)	2012	I	296 (122 NSCLC)	PD1	Nivolumab	>1 line	0.3–10 mg/kg/2 w	Squamous 33% Non-squamous12%	24 w: 22–33%	–
Brahmer et al. (14)	2012	I	207 (75 NSCLC)	PDL1	BMS-936559	>1 line	0.3–10 mg/kg/2 w	10.2%	24 w : 31%	–
Gettinger et al. (15, 16)	2015 2018	I	129	PD1	Nivolumab	>1 line	1–10 mg/kg/2 w	Squamous 17% Non-squamous18%	2.3 m	9.9 m 5 y16%
Garon et al. (17) Hui et al. (18)	2015 2017	I	495 (101 1st line)	PD1	Pembrolizumab	Any line	2–10 mg/kg/3 w 10 mg/kg/2w	19.4% (26.7%)	3.7 m (6.2 m)	12 m (22.1 m)
Rizvi et al. (19)	2015	II	117 squamous	PD1	Nivolumab	>2 lines	3 mg/kg/2 w	14.5%	1.9 m	8.2 m
Garassino et al. (20)	2018	II	444	PDL1	Durvalumab	>2 lines	10 mg/kg/2 w	3.6–30.9%	1.9–3.3 m	9.9–13.3 m
Antonia et al. (21)	2016	Ib	102	PDL1 CTLA4	Durvalumab Tremelimumab	Any line	3–10–15–20 mg/kg/4 w; 10 mg/kg/2 w 1–3–10 mg/kg/4 w (6 doses), then every 12 weeks (3doses)	17%	–	–
Hellman et al. (22)	2017	I	78	PD1 CTLA4	Nivolumab Ipilimumab	1st line	Nivo 3 mg/kg/2 w + ipi 1 mg/kg/12 w Nivo 3 mg/kg/2 w + ipi 1 mg/kg/6w	47% 38%	8.1 m 3.9m	1 y– 1 y69%
Kanda et al. (23)	2016	Ib	24	PD1	Nivolumab + CT	1st line or ≤ 2	10 mg/kg/3 w	16.7–100%	3.15 m – NR	–
Liu et al. (24)	2018	Ib	76	PDL1	Atezolizumab + CT	1st line	15 mg/kg/3 w (1,200 mg/3 w)	36–68%	5.7–8.4 m	12.9–18.9 m
Forde et al. (25) Bott et al. (26)	2018	I	21	PD1	Nivolumab neoadjuvant before surgery	1st line	3 mg/kg/2 w twice	10% Major pathological response45%		–
Yi et al. (27) Yang et al. (28)	2017 2018	II	24	CTLA4	CDDP/CBDCA-PTX-ipilimumab neoadjuvant before surgery	1st line	10 mg/kg cycles 2–3 of chemotherapy	58%	–	29.2 m

*RR, response rate; mPFS, median progression free survival; MST, median survival time; NSCLC, non-small cell lung cancer; w, week; m, months; y, year; CT, chemotherapy; NR, not reached; CDDP, cisplatin; CBDCA, carboplatin; PTX, paclitaxel*.

**Table 2:** Randomized trials comparing antiPD1/PDL1 antibody vs. salvage chemotherapy.

**References**	**Histology**	**Treatment**	***N* pts**	**RR**	***p***	**mPFS**	***p***	**MST**	***p***
Brahmer et al. (29) Horn et al. (30) Vokes et al. (31)	Squamous	Nivolumab	135	20%	0.008	3.5 m	<0.001	9.2 m 2 y 23% 3 y 16%	<0.001
		Docetaxel	137	9%		2.8 m		6.0 m 2 y 8% 3 y 6%	
Borghaei et al. (32) Horn et al. (30) Vokes et al. (31)	Non-squamous	Nivolumab	292	19%	0.02	2.3 m	0.39	12.2 m 2 y 29% 3 y 18%	0.002
		Docetaxel	290	12%		4.2 m		9.4 m 2 y 16% 3 y 9%	
Herbst et al. (33)	NSCLC PDL1≥1%	Pembrolizumab 2 mg/kg	345	–	–	3.9 m	0.07 0.004	10.4 m	0.0008 <0.0001
		Pembrolizumab 10 mg/kg	346	–		4.0 m		12.7 m	
		Docetaxel	343	–		4.0 m		8.5 m	
Fehrenbacher et al. (34)	NSCLC	Atezolizumab	144	15%	–	2.7 m	NS	12.6 m	0.04
		Docetaxel	143	15%		3.0 m		9.7 m	
Rittmeyer et al. (35)	NSCLC	Atezolizumab	425	14%	–	2.8 m	NS	13.8 m	0.0003
		Docetaxel	425	13%		4.0 m		9.6 m	
Barlesi et al. (36)	NSCLC	Avelumab	396 (264 PDL1+)	15% (19)	0.055 (0.01)	2.8 m (3.4)	0.95 (0.53)	10.5 m (11.4)	0.12 (0.16)
		Docetaxel	396 (265 PDL1+)	11% (12)		4.2 m (4.1)		9.9 m (10.3)	

*RR, response rate; mPFS, median progression free survival; MST, median survival time; NSCLC, non-small cell lung cancer; m, months; NS, not significant*.

**Table 3:** Randomized trials assessing first-line immunotherapy in stage IV NSCLC.

**References**	**Histology**	**Treatment**	***N* pts**	**RR**	***p***	**mPFS**	***p***	**MST**	***p***
**Immunotherapy monotherapy vs. chemotherapy**									
Carbone et al. (37)	NSCLC (PD-L1 ≥ 1%)	Nivolumab	211	26%	NS	4.2 m	0.25	14.4 m	NS
		Platinum doublet	212	33%		5.9 m		13.2 m	
Reck et al. (38)	NSCLC (PD-L1>50%)	Pembrolizumab	154	44.8%	–	10.3 m	<0.001	HR 0.60 (IC 95% 0.41–0.89)	0.005
		Platinum doublet	151	27.8%		6.0 m			
Mok et al. (39)	NSCLC (PD-L1 ≥ 1%)	Pembrolizumab	637	27%	–	5.4 m	NS	16.7 m	0.0018
		Platinum doublet	637	27%		6.5 m		12.1 m	
Hellmann et al. (40)	NSCLC (PD-L1 ≥ 1% + high TMB)	Nivolumab + ipilimumab	139	45.3%	–	7.2 m	<0.001	–	–
		CDDP/CBDCA-PEM or GEM	160	26.9%		5.5 m		–	
Hellmann et al. (41)	NSCLC (PD-L1 ≥ 1%)	Nivolumab + ipilimumab	396	35.9%	–	–	–	17.1 m	0.007
		CDDP/CBDCA-PEM or GEM	397	30%		–		14.9 m	
Rizvi et al. (42)	PDL1>25%	Durvalumab	163			4.7 m		16.3 m	0.036
		Durvalumab + Tremelimumab	163			3.9 m		11.9 m	0.202
		Platinum-PEM or GEM or PTX	162			5.4 m		12.9 m	
Antonia et al. (43, 44)	Stage III NSCLC^$^	Durvalumab	473	28.4%	<0.001	17.2 m	<0.001	NR	
		Placebo	236	16%		5.6 m		28.7 m	0.0025
**Immuno-chemotherapy vs. chemotherapy**									
Langer et al. (45)^*^	NSCLC	CBDCA-PEM-Pembrolizumab	60	55%	0.0016	13.0 m	0.01	HR 0.90 (IC 95% 0.42–1.91)	NS
		CBDCA-PEM	63	26%		8.9 m			
Paz-Ares et al. (46)	Squamous	CBDCA-(nab)PTX-Pembrolizumab	278	57.9%	–	6.4 m	<0.001	15.9 m	<0.001
		CBDCA-(nab)PTX	281	38.4%		4.8 m		11.3 m	
Gandhi et al. (47)	Non-squamous	CDDP/CBDCA-PEM-Pembrolizumab	410	47.6%	<0.001	8.8 m	<0.001	NR	<0.001
		CBDCA-PEM	206	18.9%		4.9 m		11.3 m	
Socinski et al. (48)	Non-squamous	CBDCA-PTX-Beva-Atezolizumab	400	63.5%	–	8.3 m	<0.001	19.2 m	0.02
		CBDCA-PTX-Beva	400	48%		6.8 m		14.7 m	
Lynch et al. (49)^*^	NSCLC	CBDCA-PTX	66	14%	–	4.6 m		8.3 m	
		CBDCA-PTX-concurrent ipilimumab	70	21%		5.5 m	0.13	9.7 m	0.48
		CBDCA-PTX-phased ipilimumab	68	32%		5.7 m	0.05	12.2 m	0.23
Papadimitrakopoulou (50)	Non squamous	CBDCA/CDDP-PEM-Atezolizumab	292			7.6 m	<0.0001	18.1 m	0.08
		CBDCA/CDDP-PEM	286			5.2 m		13.6 m	
Cappuzzo et al. (51)	Non-squamous	CBDCA-nabPTX-Atezolizumab	451	49.2%	–	7.0 m	<0.0001	18.6 m	0.033
		CBDCA-nabPTX	228	31.9%		5.5 m		13.9 m	
Jotte et al. (52)	Squamous	CBDCA-nabPTX-Atezolizumab	343			6.3 m	0.0001	14.0 m	0.69
		CBDCA-nabPTX	340			5.6 m		13.9 m	
Govindan et al. (53)	Squamous	CBDCA-PTX-ipilimumab	388	44%	–	5.6 m	0.07	13.4 m	0.25
		CBDCA-PTX	361	47%		5.6 m		12.4 m	

*RR, response rate; mPFS, median progression free survival; MST, median survival time; NSCLC, non-small cell lung cancer; m, months; NS, not significant; CBDCA, carboplatin; PEM, pemetrexed; HR, hazard ratio; CI, confidence interval; PTX, paclitaxel; CDDP, cisplatin; NR, not reached; TMB, tumor mutation burden; GEM, gemcitabine; Beva, bevacizumab*.

* *Randomized phase II*.

$ *Non-progressing stage III NSCLC after concomitant chemoradiotherapy*.

**Table 4:** Summary of selected meta-analyses on immunotherapy activity in stage IV NSCLC.

**References**	**Objective(s)**	***N* studies**	***N* pts**	**Main results**
Zhao et al. (54)	Nivolumab ORR, 1-year OS, PFS at 24 weeks, any-grade AEs, grade 3–4 AE	20 (non-comparative and RCT)	3,404	ORR 18%, 24 weeks PFS 42%, 1-year OS 45%, any-grade AEs 61%, grade 3–4 AE 12%
Khunger et al. (55)	Comparison of monotherapy antiPD1/PDL1 in 1st line vs. 2nd line	17 (Phase I-III)	4,557	ORR 1st line 30.2% vs. 2nd line 20.1% (*p* = 0.02) Median PFS 25.51w vs. 13.96 (*p* = 0.2)
Li et al. (56)	CR rate with ICI vs. CT	9 (RCT)	4,803	ICI 1.5% (95%CI: 0.8–3.0) vs. CT 0.7% (95% CI: 0.4–1.2) (RR 2.89, 95% CI: 1.44–5.81, *p* = 0.003) - Atezolizumab (RR 3.26, *p* = 0.01) - Nivolumab (RR 4.83, *p* = 0.042) - Pembrolizumab (RR 1.005, *p* = NS) - Ipilimumab (RR 0.465, *p* = NS) - First-line (RR 2.39, 95% CI: 1.08–5.3, *p* = 0.032) - Second-line (RR 4.99, 95% CI: 1.10–22.66, *p* = 0.038)
Lee et al. (57)	OS in ICI vs. docetaxel (2nd line)	5 (RCT)	3,025	HR 0.69 (95%CI, 0.63–0.75; *p* < 0.001) Subgroups: - EGFR wild-type: HR 0.67 (*p* < 0.001) vs. EGFR mutant: HR 1.11 (*p* = 0.54) - KRAS mutant: HR 0.65 (*p* = 0.03) vs. KRAS wild-type: HR 0.86 (*p* = 0.24). - No effect of smoking status, performance status, age, histology, or sex
Marur et al. (58)	Effect of age on OS in ICI vs. docetaxel (2nd line)	4 (RCT)	2,824	HR for os: <65 years 0.71 (95% CI 0.63, 0.80), ≥65 years 0.66 (95% CI 0.57, 0.76), ≥70 years 0.67 (95% CI 0.55, 0.82), ≥75 years 0.81 (95% CI 0.58, 1.13)
Addeo et al. (59)	OS and PFS of ICI-CT vs. CT (1st line)	8 (RCT)	4,646	OS HR 0.74 (95% CI 0.64–0.87; *p* = 0.0002) PFS HR 0.61 (95% CI 0.56–0.66; *p* < 0.00001)
Chen et al. (60)	OS, PFS, and RR of ICI (+/– CT) vs. CT (1st line)	12 (RCT)	8,384	OS HR 0.77 (95% CI 0.64–0.91, *p* = 0.003) PFS HR 0.66 (95% CI 0.57–0.77, *p* < 0.00001) ORR RR 1.97 (95% CI 1.25–3.13, *p* = 0.004)
Conforti et al. (61)	Effect of gender on ICI activity (1st line)	8 (RCT)	4,923	Pooled ratio of OS HR (men vs. women) 1.56 (95% CI 1.21–2.01)
Kim et al. (62)	Comparative efficacy of 1st line pembrolizumab	4 (RCT)	2,754	PFS: Pembrolizumab-CT > Pembrolizumab (*p* = 0.048) (PDL1≥50%) OS: Pembrolizumab-CT = Pembrolizumab (*p* = 0.485) (PDL1≥50%)

*ORR, objective response rate; OS, overall survival; PFS, progression-free survival; AE, adverse effects; CT, chemotherapy; RCT, randomized controlled trials; w, weeks; CR, complete response; ICI, immune checkpoint inhibitor; RR, relative risk; CI, confidence interval; NS, not significant; HR, hazard ratio; OR, odds ratio*.

**Table 5:** Summary of selected meta-analyses on indirect comparison between immunotherapies in stage IV NSCLC.

**References**	**Objective(s)**	***N* studies**	***N* pts**	**Main results**
Wang et al. (63)	Comparative efficacy of 1st line ICI in wild-type NSCLC (nivolumab, atezolizumab, pembrolizumab)	9 (RCT)	5,504	Survival better with pembrolizumab plus chemotherapy than with pembrolizumab alone and other chemo-immunotherapy regimens
Frederickson et al. (64)	Comparative efficacy of 1st line ICI in wild-type non-squamous NSCLC (pembrolizumab, atezolizumab, standard CT)	22 (RCT)	11,178	Pembrolizumab-platinum doublet has 95.6% probability to be the best treatment for OS Pembrolizumab-platinum doublet has 67.3% probability to be the best treatment for PFS
Passiglia et al. (65)	Comparative efficacy of 2nd line ICI (nivolumab, atezolizumab, pembrolizumab)	5 (RCT)	3,355	ORR: nivolumab = pembrolizumab, nivolumab > atezolizumab, pembrolizumab > atezolizumab PFS: nivolumab = pembrolizumab = atezolizumab OS: nivolumab = pembrolizumab = atezolizumab AE: nivolumab better than pembrolizumab and atezolizumab, pembrolizumab = atezolizumab

*ICI, immune checkpoint inhibitor; NSCLC, non-small cell lung cancer; CT, chemotherapy; OS, overall survival; PFS, progression-free survival; ORR, objective response rate; AE, adverse events*.

### Phase I-II Studies

Ten phase I and 3 phase II studies were retrieved, corresponding to 14 different publications. Their main characteristics are summarized in Table 1. Eight assessed immunotherapy monotherapy, two a combination of antiPD1 or antiPDL1 and antiCTLA4 and the last three evaluated the combination of immunotherapy and chemotherapy. Except four, all studies were including pre-treated patients.

In this sometimes heavily pre-treated population, interesting response rates were noted, ranging from 3.6% to up to 100% in a small group receiving nivolumab and chemotherapy. Median progression-free survival (PFS), probably not the best endpoint to be considered in this type of trial, was at least similar to what can be observed with conventional salvage chemotherapy, between 2 and 3 months. However, increased PFS was reported in 4 studies, from 5.7 months up to 8.4 months (17, 18, 22, 24). Data on overall survival (OS) showed surprisingly long median survival time (MST), above 8 months. An updated analysis of a phase I trial (15, 16) reported on 5-year survival rates. Among 129 advanced NSCLC receiving nivolumab for up to 96 weeks, of whom 80 patients had at least two previous chemotherapy lines, a 16% 5-year survival rate was observed. Among these 16 patients, 12 were alive at 5 years without any evidence of tumor recurrence. No difference in 5-year survival rates was noted according to histology (squamous 16% and non-squamous 15%). There seems to exist a potential relationship between PDL1 expression and 5-year survival rates, which was higher for tumors with PDL1≥50% (43%) than in those with low/no expression (23%/20%). No definite conclusion can be drawn from these results as this analysis was retrospective and no formal comparison was available in the manuscript. In the same way, the KN001 study including 550 patients receiving pembrolizumab were updated and showed MST of 22.3 and 10.5 months and 5-year survival rates of 23.2 and 15.5%, for treatment naïve and previously treated NSCLC, respectively (66, 67).

Most of the available studies were performed in advanced/metastatic recurrent NSCLC. Two studies were done in the neoadjuvant setting (25–28). In a phase I study (25, 26), 21 eligible patients with stage IB-IIIA resectable NSCLC received two doses of nivolumab (3 mg/kg) on days 28 and 14 before surgical resection. Complete resection was achieved in 20 patients. Despite a minimal 10% response rate based on RECIST criteria, major pathological response (<10% viable tumor cells) was reported in 40% of the patients. No unexpected adverse event and no operative mortality were observed (26). The primary objective of the phase II study (27, 28) was “to assess whether the appearance of T cells activated against select tumor-associated antigens (TAA) increased from baseline following treatment with ipilimumab” and this endpoint was not met. Twenty-four stage IIA-IIIA NSCLC received 3 cycles of induction cisplatin or carboplatin plus paclitaxel with ipilimumab (10 mg/kg) during cycles 2 and 3. Eleven were not resected because of persistent N2 disease, inoperability of the patient or cancer progression. Grade 3–4 adverse events were experienced in 46% of the patients while immune-related adverse events were more prominent than with nivolumab: grade 2 pneumonitis (4%), grade 3 adrenal insufficiency (17%), and diarrhea/colitis (38%). Thirteen patients had a complete resection and perioperative complications appeared of similar magnitude than expected for resected stage II-IIIA NSCLC.

Overall, phase I-II studies not only allowed adequate dose finding for further trials but also sent major signals of activity based on response rate and PFS in metastatic diseases in both the immunotherapy monotherapy or combination setting. Further, impressive survival results were reported that needed confirmation in larger prospective studies. The first data on induction immunotherapy demonstrated the feasibility of antiPD1 monotherapy in terms of safety and activity while antiCTLA4 in combination with induction chemotherapy demonstrated increased toxicity and questionable activity.

### Randomized Trials Comparing antiPD1 or antiPDL1 to Salvage Chemotherapy

Ten publications corresponding to 6 randomized trials were retrieved. Main inclusion criteria were similar across the trials: performance status 0–1, no activating mutation (patients with oncogenic driver mutation, essentially activating EGFR mutations, could be included provided they exhausted targeted therapies), no auto-immune disease (at the exception of psoriasis and Graves' disease that were generally allowed), no interstitial lung disease, no systemic steroids (maximal authorized dose of prednisone between 7.5 and 10 mg/day), and no untreated and progressive brain metastases. The comparator was also similar in all 6 trials with docetaxel given at 75 mg/m^2^ every 3 weeks, and the activity of this chemotherapy was similar across the trials and as expected based on previous clinical trials (68). The data are summarized in Table 2.

Nivolumab was tested in two phase III trials, including either squamous (29–31) or non-squamous NSCLC (30–32). In the squamous population (CheckMate 017), all endpoints significantly improved with nivolumab in comparison with docetaxel: response rate (20 vs. 9%), PFS (median 3.5 vs. 2.8 months), and OS (median 9.2 vs. 6 months). In non-squamous patients (CheckMate 057), response rate was superior in the nivolumab group while there was no difference in PFS. Also, OS significantly improved with nivolumab. No definite conclusions can be drawn for a predictive role of PDL1 on further nivolumab effectiveness. Three updated survival analyses are available. They confirmed increased 2 and 3-year survival rates, significantly better in the immunotherapy groups. A combined analysis of the two trials (69) showed a 14% 4-year survival rates with nivolumab while only 5% of the patients were alive in the docetaxel group. Despite that nivolumab was also superior in the PDL1 negative subgroup (9 vs. 4%), the magnitude of effect was higher in the PDL1≥1% NSCLC (20 vs. 4%). These data are confirming the 5-year survival rates observed in the phase I studies (15). Further, tolerance was better with nivolumab than with docetaxel. Zhao et al. (54) performed a meta-analysis on nivolumab activity in salvage setting after platinum-based regimen. They included 20 non-comparative (*n* = 17) and randomized (*n* = 3) clinical trials corresponding to 3,404 patients. After excluding studies with high heterogeneity, response rate was 18% (95% CI 15–20%) with corresponding 24-weeks PFS of 42% (95% CI 37–48%) and 1-year OS of 45% (95% CI 40–50%). Cumulative grade 3–4 adverse events rate was 12% (95% CI 9–16%). A positive association with PDL1 expression and response rate was noted.

Usually, patients in randomized trials are selected populations with few poor prognostic factors, younger age and less co-morbidities. The access to real life data is of importance for extrapolating data from clinical trials to routine practice. In this way, the Italian expanded access program (70) confirmed the results of the randomized trials. Response rate was 18% and no difference was noted across age subgroups (<65 years, 66–74 years, >75 years). Also, median PFS were similar: 4.2 months (overall population), 4 months (<65 years), 4.5 months (66–74 years), 3.2 months (>75 years). Except in the group >75 years (median OS 5.8 months), survival rates were similar (overall 7.9 months, <65 years 8.6 months, and 66–74 years 8 months). No difference was statistically significant. Grade 3–4 adverse event incidence was low (3–9% according to subgroups).

The second antiPD1, pembrolizumab, was tested in a population of NSCLC with PDL1 expression above 1% (33). Two dose levels of pembrolizumab were administered, 2 and 10 mg/kg every 3 weeks. No data on response rate were presented. At each dose level, pembrolizumab significantly increased PFS and OS over docetaxel. Pembrolizumab effectiveness appeared more pronounced in high PDL1 expressors (≥50%) while no interaction test was performed precluding formal conclusion. Subgroup analyses demonstrated no statistically significant survival difference in the squamous population but these analyses remained of exploratory value. As for nivolumab, the toxicity profile was better for immunotherapy than for chemotherapy.

Two randomized trials compared atezolizumab to docetaxel. The first was a randomized phase II study (34) with OS as primary endpoint. Atezolizumab demonstrated no impact on response rate and PFS but significantly improved OS. These results were confirmed in the phase III trial (OAK) (35). Response rate and PFS were similar in both arms but OS significantly increased in the atezolizumab arm. A secondary analysis according to PDL1 status showed that superiority of atezolizumab was conserved in any subgroup while the magnitude of benefit increased in parallel with PDL1 expression. As different efficacy and toxicity profiles with chemotherapeutic drugs and increased driver mutation incidence were previously reported in Asian patients, it could be of interest confirming the same effectiveness of ICI immunotherapy in this specific population. In a separate sub-group analysis of 64 patients, the effectiveness of atezolizumab was confirmed in Asian patients (71).

On the opposite of these three drugs, the antiPDL1 avelumab, in the same disease setting did not demonstrate any superiority over docetaxel whatever the endpoint (response rate, PFS or OS) or in the PDL1+ subgroup, at the exception of a small increase in response rate (36). Despite no improvement over docetaxel, avelumab showed activity in this population. The authors explaining the difference with the previous phase III trials have suggested some explanations. More checkpoint inhibitors were used after docetaxel cessation: 26 vs. 17% in the azetolizumab study (35) or 13% in the pembrolizumab trial (33). Also, there were differences in biomarker evaluation (different antibody, other cut-off defining high PDL1 tumor proportion score), some patients' characteristics (more Asian patients) and maybe, drug characteristics (antiPDL1 instead of antiPD1 while atezolizumab trials were positive).

Different meta-analyses added some useful information to the field. Khunger et al. (55), including phase I to III data showed an overall response rate of immunotherapy monotherapy, antiPD1 and antiPDL1 combined, of 20.1% (95% CI 17.5–22.9%) lower than for patients receiving immunotherapy in first-line (30.2%, 95% CI 22.7–38.2%). However, they did not find any significant difference in terms of PFS frontline vs. second-line (median PFS 25.5 vs. 13.96 weeks; *p* = 0.2). More pneumonitis occurred when immunotherapy was proposed frontline (4.9 vs. 3%; *p* = 0.03). Further, the chance to obtain a complete response was increased with immunotherapy compared to chemotherapy [Relative Risk (RR) 4.99, 95% CI 1.10–22.66; *p* = 0.038] (56). A meta-analysis of 5 randomized trials (57) confirmed the superiority of antiPD1/PDL1 on docetaxel (HR for OS 0.69, 95% CI 0.63–0.75; *p* < 0.001). They found that checkpoint inhibitors prolonged survival in EGFR wild type and KRAS mutant patients but no effect of smoking status, performance status, age, histology, or sex was documented.

The effect of age was investigated in a meta-analysis of 4 randomized trials (58). No impact of age on OS could be demonstrated. The HR was 0.71 (95% CI 0.63, 0.80) in younger patients (<65 years). The HR for patients ≥65 years, ≥70 years, and ≥75 years were 0.66 (95% CI 0.57, 0.76), 0.67 (95% CI 0.55, 0.82), and 0.81 (95% CI 0.58, 1.13). The corresponding values for median OS (antiPD-1/PD-L1 vs. docetaxel) were, respectively, 14.5 and 8.8 months (<65 years), 14.2 and 9 months (≥65 years), 14.1 and 9.2 months (≥70 years), and 14.7 and 9.5 months (≥75years). The analysis suggested a reduced rate of grade 3–4 toxic events in aged patients (≥75years).

### Randomized Trials Assessing First-Line Immunotherapy in Stage IV NSCLC

According to the impressive results at short and long-term of antiPD1/PDL1 therapies for recurrent NSCLC, these drugs were further tested in first-line in stage IV NSCLC. Two approaches were developed, immunotherapy monotherapy or combined chemo-immunotherapy, both tested against standard platinum-based regimens. The data of the phase III randomized trials are summarized in Table 3.

#### Immunotherapy Monotherapy vs. Chemotherapy

Nivolumab, the first antiPD1 antibody demonstrating a survival advantage for salvage chemotherapy was tested in a population of stage IV NSCLC harboring PDL1≥1%, including both squamous and non-squamous histologies (37). Comparators were platinum doublets. The primary efficacy analysis was restricted to the population with PDL1≥5%. No difference was observed whatever considering response rate, PFS or OS. An exploratory analysis in patients with PDL1≥50%, with imbalance in the number of patients and in sex between both groups, also did not show any advantage of nivolumab. Another exploratory analysis suggested that high tumor-mutation burden (TMB) was associated with increased response rate (47 vs. 28%) and PFS (median 9.7 vs. 5.8 months) in the nivolumab group while there was no impact on OS. Reduced all grade and grade 3–4 toxicity was associated with nivolumab. A complex phase III trial (Checkmate 227) tested in different PDL1 strata, nivolumab-based regimens vs. chemotherapy. The first published data (40) focused on the comparison between the nivolumab-ipilimumab combination and chemotherapy (cisplatin/carboplatin plus pemetrexed or gemcitabine), in patients with high TMB (≥10 mutations per megabase tested with the FoundationOne CDx assay). The authors presented an increased response rate (45%, 95% CI 36.9–54% vs. 26.9%, 95% CI 20.2–34.4%) and PFS (median 7.2 vs. 5.5 months; HR 0.58; 97.5% CI 0.41–0.81, *p* < 0.001). The impact of the nivolumab-ipilimumab combination on PFS was observed both in PDL1 <1% and above 1%. Despite similar adverse events rates in both arms, more treatment discontinuations were observed in the immunotherapy arm. Of exploratory value, the authors provided information on improved PFS with nivolumab-ipilimumab (HR 0.83, 95% CI 0.72–0.96) in the whole population, irrespective of the TMB and PDL1 status. However, no statistically significant difference in PFS was found in the low TMB group (HR 1.07, 95% CI 0.84–1.35). Finally, in the patients with high TMB (>10 mutations per megabase) and a PDL1≥1% receiving nivolumab alone, no difference with chemotherapy in terms of PFS was reported (HR 0.95, 95% CI 0.61–1.48). These data were completed by a second publication (41). Survival in the nivolumab-ipilimumab arm was significantly improved in comparison with chemotherapy arm with respective median duration of 17.1 months (95% CI 15.2–19.9 months) and 13.9 months (95% CI 12.2–15.1 months). The same difference was observed in the PDL1≥1% subgroup (MST 17.1 months, 95% CI 15.0–20.1 months vs. 14.9 months, 95% CI 12.7–16.7 months; *p* = 0.007) and in the PDL1 <1% tumors (MST 17.2 months, 95% CI 12.8–22.0 months vs. 12.2 months, 95% CI 9.2–14.3 months) without formal comparison according to the study design. In a secondary analysis, nivolumab-ipilimumab was compared to nivolumab monotherapy in tumors with PDL1≥1% and ≥50%. A slight increase in 2-year survival and response duration was observed with the combination (no *p*-value provided). While a slight increase in response rate was observed in the nivolumab-chemotherapy regimen (tumors with PDL1≥1%), 2-year survival and response duration favored the immunotherapy doublet (no formal statistical comparison). PDL1 status and TMB, alone or in combination did not have any predictive value for the antitumoural effectiveness. The authors did not demonstrate increased grade 3–4 toxicity with thenivolumab-ipilimumab combination in comparison with chemotherapy.

Two trials assessed the efficacy of pembrolizumab in NSCLC in which all histological types were eligible. In the first trial (Keynote 024) (38), only tumors harboring high PDL1 expression (≥50%) were considered. Pembrolizumab (200 mg every 3 weeks for 35 cycles) was compared to platinum-based regimens with pemetrexed, gemcitabine or paclitaxel. Despite high crossover rates (43.7%) to pembrolizumab after progression on chemotherapy, increased response rate and statistically significant better PFS and OS were noted in the pembrolizumab arm. Severe grade 3–5 adverse events occurred twice more in the chemotherapy arm (53.3 vs. 26.6%). The second trial (Keynote 042) (39), had a similar design to the Keynote 024 but as well included tumors with lower PDL1 (≥1%). As stated in the manuscript, “In the original protocol, written in 2014, the primary endpoint was overall survival in patients with a PD-L1 TPS of 50% or greater and secondary endpoints were overall survival in patients with a PD-L1 TPS of 1% or greater and progression-free survival in patients with a TPS of 50% or greater and of 1% or greater” (39). However, according to the results of the Keynote 010 and the CheckMate 026 studies, the primary endpoint was amended to OS in three groups according to the PDL1 status (≥50%, ≥20%, and ≥1%). OS significantly increased with pembrolizumab in all 3 planned groups with HR 0.69 (*p* = 0.0003), HR 0.77 (*p* = 0.002), and HR 0.81 (*p* = 0.0018) in the “≥50%,” “≥20%,” and “≥1%” groups, respectively. However, when the analysis was restricted to the tumors with PDL1 between 1 and 49%, no survival advantage was noted (HR 0.92; 95% CI 0.77–1.11). Based on these analyses, we can extrapolate that superiority of pembrolizumab monotherapy is restricted to tumors with PDL1≥50% and this schedule cannot be recommended yet for tumors with lower PDL1 expression. No significant difference in terms of response rate and PFS was observed and the favorable toxicity profile of pembrolizumab was confirmed.

Besides antiPD1, one antiPDL1 was tested both in stage IV and for adjuvant therapy in stage III NSCLC. The first trial, MYSTIC, is only available in its abstract format (42). NSCLC patients with PDL1≥25% were randomized between 3 arms: durvalumab alone, a combination of durvalumab and tremelimumab or a platinum-based doublet with pemetrexed, gemcitabine or paclitaxel. First data showed similar PFS (no statistical comparison was provided). Improved survival was observed with durvalumab vs. chemotherapy but the immunotherapy combo was not superior to chemotherapy. Final results are awaited.

The PACIFIC trial (43, 44) randomly assigned patients with stage III NSCLC not progressing after concomitant chemo-radiotherapy to adjuvant durvalumab or placebo for 1 year. Despite many criticisms based e.g., on the lack of information concerning the initial locoregional treatment, important improved PFS and OS were found with adjuvant immunotherapy. *Post-hoc* analyses suggested that patients with tumors having no PDL1 expression derived no benefit from adjuvant durvalumab (PFS HR 0.73, 95% CI 0.48–1.11 and OS HR 1.36, 95% CI 0.79–2.34). Toxicity profile was as expected with antiPDL1 antibodies despite previous thoracic irradiation, mainly grade 3–4 pneumonitis (4.8 vs. 2.6%) while grade 3–4 radiation pneumonitis rates were similar (1.3% in both arms). According to these data, the European Medicine Agency (EMA) agreed for marketing authorization with the following indication: “Imfinzi as monotherapy is indicated for the treatment of locally advanced, unresectable non-small cell lung cancer (NSCLC) in adults whose tumors express PD-L1 on ≥1% of tumor cells and whose disease has not progressed following platinum-based chemoradiation therapy” (72). This decision not including tumors with PDL1 <1% was made on an unplanned *post-hoc* exploratory analysis requested by the EMA and is not based on the intent-to-treat population. In a position paper, the authors presented various arguments not supporting the EMA decision: heterogeneity of the PD-L1 expression inside the tumor, PDL1 expression influenced by radiotherapy and/or chemotherapy limiting the value of pre-treatment data, lack of available tissue samples (37%) and of guarantee that all confounding factors are well-balanced (73).

#### Combined Chemotherapy-Immunotherapy vs. Chemotherapy

Another question was to define if the addition of immunotherapy to chemotherapy can improve the efficacy of standard chemotherapy. This could be of importance as it was noted in previous trials comparing immunotherapy monotherapy to chemotherapy that a number of patients on immunotherapy had a worse evolution and rapid progression at first evaluation (38, 39). It must be emphasized that we do not have direct comparison between immunotherapy monotherapy and combined immunotherapy and chemotherapy. Currently, we cannot answer the question of the interest of the reverse sequence, adding chemotherapy to immunotherapy.

The first phase II randomized trial investigated carboplatin-pemetrexed plus or minus pembrolizumab (45). Despite higher response rate (primary endpoint) and PFS, no significant impact on survival was associated with the triplet regimen. A slight increase in toxicity was observed in the triplet, including immune-related reactions. Two other phase III trials with similar design were performed in squamous [comparator carboplatin-(nab)paclitaxel] (46) and non-squamous histologies (comparator cisplatin/carboplatin-pemetrexed) (47). In both, the triplet regimen improved all endpoints: response rate, PFS and OS. The benefit of adding pembrolizumab to chemotherapy was maintained across all PDL1 subgroups, also in tumors without any PDL1 expression although with lower benefit. A supplemental toxicity, including immune-related reactions, was noted but remained manageable. It must be noted that there was an increase in renal toxicity that should be considered and followed for cisplatin administration in combination with pembrolizumab. In the Gandhi trial (47), no difference in terms of OS, PFS, and RR was observed according to the platinum compounds, cisplatin or carboplatin.

The antiPDL1, atezolizumab was tested in a complex program with four presented trials. Two trials tested carboplatin-nabpaclitaxel plus or minus atezolizumab separately in squamous (52) and non-squamous (51) histologies. The two trials are available in their abstract format. In both, better PFS was associated with the triplet. However, the positive impact on OS was only reported in non-squamous histology (51). This maybe could be explained by unexpected survival rates in the comparator arm as observed MST in the chemotherapy-immunotherapy arm compared well with the other RCT. Two phase III trials used a platinum-pemetrexed doublet in non-squamous NSCLC. Papadimitrakopoulou (50) presented in abstract a statistically significant positive association of the addition of atezolizumab to platinum-pemetrexed on PFS (*p* < 0.0001) while the OS superiority of the triplet did not reach the statistical significance. The last phase III trial (48) is a three-arm study comparing carboplatin-paclitaxel-atezolizumab (ACP), carboplatin-paclitaxel-bevacizumab (BCP) and a quadruple combination of carboplatin-paclitaxel-bevacizumab and atezolizumab (ABCP). The primary endpoints were PFS in the wild-type (WT) genotype population and in the WT population with high expression of an effector T-cell (Teff). In the publication (48), only the comparison between the ABCP and BCP arms was provided. PFS was significantly longer in the ABCP arm whatever regarding the WT population (median 8.3 vs. 6.8 months, *p* < 0.001) and in the Teff one (median 11.3 vs. 6.8 months, *p* < 0.001), regardless of PDL1 status. The same statistically significant results were observed in the patients with EGFR mutation (and a few with ALK translocation) previously receiving targeted therapy (median PFS 9.7 vs. 6.1 months), KRAS mutation (median PFS 8.1 vs. 5.8 months), and in case of liver metastases (median PFS 7.4 vs. 4.9 months). The authors presented only interim analysis for OS in the WT population, with significant superiority of ABCP. Also a slight increase in adverse events was noted in the ABCP regimen.

The last two randomized trials tested the addition of the antiCTLA4 ipilimumab to carboplatin-paclitaxel. Initially, a randomized phase II study (49) evaluated the best mode of ipilimumab administration, phased (with the last 4 doses of chemotherapy) or concurrent (with the first 4 doses of chemotherapy). The phased ipilimumab had the best response rate, PFS and OS results. No increase in toxic events was observed except for immune-related effects, as expected. The phased schedule was chosen for the phase III (53). No significant effect of ipilimumab was demonstrated whatever considering response rate, PFS or OS. In exploratory analyses, no subgroup showed any advantage for ipilimumab. Serious adverse events occurred more frequently with the antiCTLA4 antibody. On the basis of the current available evidence, antiCTLA4 antibodies have limited role in the treatment of advanced NSCLC.

Different meta-analyses summarized the activity of immunotherapy monotherapy or combined chemo-immunotherapy vs. immunotherapy alone. Addeo et al. (59) showed that chemo-immunotherapy significantly improved OS (HR 0.74; 95% CI 0.64–0.87; *p* = 0.0002) and PFS (HR 0.61; 95% CI 0.56–0.66; *p* < 0.00001). Better OS was separately confirmed with atezolizumab (HR 0.85; 95% CI 0.76–0.94; *p* = 0.001) and pembrolizumab (HR 0.56; 95% CI 0.46–0.67; *p* < 0.00001). A significant OS improvement was noted both in negative and high PDL1 subgroups but not in low PDL1 population while PFS was statistically improved in each PDL1 strata. Chen et al. (60) showed that immune checkpoint inhibitors (ICI) (+/– chemotherapy) improved OS (HR 0.77, *p* = 0.003), PFS (HR 0.66, *p* < 0.00001) but not response rate (RR 1.97, *p* = 0.004). When the analysis was restricted to ICI monotherapy, the difference was not anymore significant for OS (HR 0.82, 95% CI 0.68–1.00, *p* = 0.06), PFS (HR 0.70, 95% CI 0.39–1.26, *p* = 0.24) at the difference of combined chemo-immunotherapy (OS: HR 0.77, 95% CI 0.64–0.91, *p* = 0.003, and PFS: HR 0.66, 95% CI 0.56–0.77, *p* < 0.00001). The positive effect on OS was confirmed in the high PDL1 expressors whatever considering immunotherapy monotherapy or combination but was restricted to the combination in the PDL1 negative subgroup. Interestingly, Chen et al. had the same observation on absence of statistically significant impact on OS in the intermediate PDL1 (1–49%) population. The effect on OS and PFS was observed both in squamous and non-squamous NSCLC with the restriction of ICI monotherapy (OS: HR 0.99, 95% CI 0.73–1.34, *p* = 0.95; PFS: HR 0.74, 95% CI 0.40–1.37, *p* = 0.34).

Currently, it is difficult answering the question of the interest adding chemotherapy to immunotherapy in tumors with high PDL1 expression and which patients benefit most from the combination. In a network meta-analysis [(62); Table 4], an indirect comparison between pembrolizumab alone and in combination with chemotherapy was performed. Whatever considering PFS and OS, pembrolizumab-containing regimens demonstrated their superiority over chemotherapy, independently of the PDL1 status. An indirect comparison between monotherapy and combination was done only in the PDL1≥50% strata. Despite better PFS when pembrolizumab was combined with a platinum-doublet, this did not translate into a survival benefit. No analysis can be done for PDL1 <50% group. Zhou et al. (74) had the same conclusion in a high PDL1 subgroup. Despite improved response rate and PFS, no significant survival improvement was observed in the pembrolizumab-chemotherapy regimens in comparison with pembrolizumab alone.

In a last meta-analysis, the authors assessed the differential activity of antiPD1/PDL1 according to gender (61). While a significant activity of chemo-immunotherapy was documented in both sexes, it seems more important in women [pooled ratio of OS HR (men vs. women) 1.56; 95% CI 1.21–2.01]. When looking at the studies testing immunotherapy alone or chemoimmunotherapy, a differential effect was observed with better activity for immunotherapy alone in men (pooled ratio 0.83; 95% CI 0.65–1.06) and for chemoimmunotherapy in women (pooled ratio 1.70; 95% CI 1.16–2.49).

#### Which Is the Best Treatment Duration?

There is no formal published comparison among different treatment durations. Globally, the question remains unresolved. Nevertheless, different information provided by the randomized trials could give us some track for future studies.

In second line, immunotherapy can be continued until disease progression, and eventually beyond in case of clinical benefit, for nivolumab (29, 32), atezolizumab (34, 35), and avelumab (36). It was capped for pembrolizumab at 24 months (33).

In first-line, no limit of administration except disease progression was provided for nivolumab (37, 40) and atezolizumab (48). Pembrolizumab was limited to 35 cycles when used either in monotherapy (38, 39) or in combination with chemotherapy (45–47).

#### Is There Any Difference Among the Different antiPD1/PDL1 Antibodies?

AntiPD1 and antiPDL1 antibodies, alone or with chemotherapy, whatever the line of treatment demonstrated significant activity in advanced NSCLC. However, no direct comparisons between the different compounds or combinations were performed. Three network meta-analyses allowing indirect comparisons are detailed in Table 5.

Wang et al. (63) confirmed that 1st line immunotherapy, either nivolumab, pembrolizumab or atezolizumab considering monotherapy or combined chemo-immunotherapy, had better efficacy than chemotherapy in reducing disease progression (HR 0.69, 95% CI 0.56–0.86; *p* = 0.001) and mortality (HR 0.74, 95% CI 0.63–0.87; *p* < 0.001), and that grade 3–5 adverse events were less pronounced with immunotherapy monotherapy (RR 0.42, 95% CI 0.35–0.51; *p* < 0.001) but were worse with combined chemo-immunotherapy (RR 1.15, 95% CI 1.04–1.27; *p* = 0.008). Using pembrolizumab alone (*P*-score 0.65) as the reference comparator, only pembrolizumab plus platinum doublet (HR 0.87, 95% CI 0.79–0.95; *P*-score 0.95) showed any significant superiority while combination including nivolumab or atezolizumab did not (HR 0.93–1.13).

Frederickson et al. (64) compared 1st line randomized trials including platinum-doublets eventually with bevacizumab, atezolizumab or pembrolizumab in non-squamous NSCLC. For OS, pembrolizumab plus chemotherapy had the highest probability to be the best regimen (95.6%) while the four-drug regimen including atezolizumab and bevacizumab was the second with a probability of 2.6%. The same conclusion was proposed for PFS with respective probabilities of 67.3 and 24.1%.

In second-line, an indirect comparison of the 3 antiPD1/PDL1 tested in randomized trials was done (65). Slight differences were observed for response rate between nivolumab or pembrolizumab and atezolizumab with respective RR 1.66 (95% CI 1.07–2.58) and 1.94 (95% CI 1.30–2.90). However, no significant difference was reported for PFS and OS data. Nivolumab seemed less toxic than the two other compounds (grade 3–5 adverse events: Relative Risk 0.41, 95% CI 0.29–0.60 and Relative Risk 0.50, 95% CI 0.35–0.72).

#### Does Immunotherapy Have an Impact on Quality of Life?

Secondary exploratory analyses from randomized trials provided information on quality of life (QoL) or PROM (patient-related outcome measure) (Table 6). In the OAK trial (75), no statistically significant difference between atezolizumab and docetaxel was observed regarding overall QoL assessed by the EORTC-QLQC30 questionnaire. However, a significant reduction in time of deterioration in physical and role functions and an increase time of deterioration for chest pain was associated with atezolizumab while no worsening was found with the antiPDL1. In the non-squamous population receiving 2nd line nivolumab (76), QoL endpoints (LCSS scales) significantly improved overall (Table 6) or for the following variables: fatigue, cough, dyspnea, hemoptysis, interference with activity level and health-related QoL with statistically significant between-arm differences at weeks 12, 24, 30, and 42. While the difference was borderline for LCSS, the same conclusions on QoL improvement after week 12 can be derived from 2nd line nivolumab in squamous NSCLC (77). Also, in first-line, immunotherapy (pembrolizumab) in high PDL1 tumors was associated with a better quality of life over time and reduced time to deterioration, overall and in most functioning and symptoms domain (78). All the data were summarized in a recently published systematic review (83). For most of the endpoints, whatever the QoL scales that were used and the underlying cancer types, no deterioration associated with immunotherapy was reported instead of better QoL overall or for physical and symptoms domains.

**Table 6:** Quality of life analyses from randomized controlled trials.

**Reference**	**Trial**	***N* patients**	**Main results**
Bordoni et al. (75)	OAK (atezolizumab vs. docetaxel)	803	HRQoL: HR 0.94 (95% CI 0.72–1.24; *p* = 0.66) TTD in physical function: HR 0.75 (95% CI 0.58–0.98; *p* = 0.03) TTD in role function: HR 0.79 (95% CI, 0.62–1.00; *p* = 0.05) TTD in chest pain: HR 0.71 (95% CI 0.49–1.05; *p* = 0.08)
Reck et al. (76)	Checkmate 057 (nivolumab vs. docetaxel in non-squamous NSCLC)	420	LCSS ABSI: HR 0.65 (95% CI 0.49–0.85; *p* = 0.002) LCSS-3-IGI : HR 0.63 (95% CI 0.48–0.82; *p* < 0.001) EQ-5D utility index : HR 0.90 (95% CI 0.69–1.17; *p* = 0.42) EQ-5D VAS : HR 0.76 (95% CI 0.59–0.98; *p* = 0.032)
Reck et al. (77)	Checkmate 017 (nivolumab vs. docetaxel in squamous NSCLC)	181	LCSS ABSI: HR 0.67 (95% CI 0.43–1.03; *p* = 0.07) LCSS-3-IGI : HR 0.57 (95% CI 0.38–0.85; *p* = 0.005) EQ-5D utility index : HR 0.55 (95% CI 0.36–0.84; *p* = 0.006) EQ-5D VAS : HR 0.59 (95% CI 0.40–0.87; *p* = 0.008)
Brahmer et al. (78)	Keynote 024 (pembrolizumab vs. platinum-CT, PDL1>50%)	299	At week 15: improvement of QLQ C30 of 7.8 points (2.9–12.8; *p* = 0.002) TTD (pembrolizumab vs. CT): median not reached vs. 5.0 months (HR 0.66, 95% CI 0.44–0.97; *p* = 0.029)

*HRQoL, health related quality of life; HR, hazard ratio; CI, confidence interval; TTD, time to deterioration; NSCLC, non-small cell lung cancer; CT, chemotherapy*.

#### Is Immunotherapy Cost-Effective?

Costs in treating NSCLC patients are dramatically increasing during the last decade, as it is also true in other cancer populations. Beyond the clear superior effectiveness of immunotherapy (plus or minus chemotherapy) on standard chemotherapy, we may question the viability of the national health systems sustaining expensive drugs in a large proportion of patients that remained administered with a palliative intent. Cost-effectiveness analyses were derived from randomized trials with pembrolizumab and atezolizumab. As reimbursement criteria and health insurances largely differ from one country to another, pharmacoeconomic analyses can only be interpreted in the specific system in which they were achieved.

Three US studies dealed with pembrolizumab, either in 1st or 2nd line (79–81). Incremental costs (in US dollars) per QALY (quality of life adjusted years) ranged from 97,621$ to 168,619$. This was considered cost-effective in the US using a threshold of 3-times gross domestic product per capita. In the PDL1≥50% subgroup, the incremental costs per QALY were 103,402$ (pembrolizumab-chemotherapy vs. chemotherapy) and 147,365$ (pembrolizumab-chemotherapy vs. pembrolizumab). The costs were higher per QALY for the combination pembrolizumab-chemotherapy in PDL1 negative NSCLC (183,529$) than for intermediate PDL1 expression (66,837$) (Table 7).

**Table 7:** Pharmacoeconomic analyses from randomized trials.

**References**	**Trial**	**Main results**
Huang et al. (79)	KEYNOTE 024 (1st line) (pembrolizumab vs. CT, US patients, PDL1≥50%)	Cost per LYG 78,344$ Cost per QALY 97,621$
Insinga et al. (80)	KEYNOTE 189 (1st line, non-squamous) (pembrolizumab-CT vs. CT, US patients, PDL1≥50%)	Cost per LYG 87,242$ Cost per QALY 104,823$
Huang et al. (81)	KEYNOTE 010 (2nd line) (pembrolizumab vs. docetaxel, US patients, PDL1≥50%)	Cost per LYG 135,552$ Cost per QALY 168,619$
Ondhia et al. (82)	OAK (2nd line) (atezolizumab vs. docetaxel, Canada)	Cost per QALY 142,074$

*LYG, life year gained; QALY, quality adjusted life years*.

The last study was conducted in Canada, using the data from the OAK trial (82). Second line atezolizumab showed incremental costs (in Canadian dollars) per QALY of 142,074$. Based on the data from a network meta-analysis, the same authors indirectly compared the cost-effectiveness of nivolumab, pembrolizumab and atezolizumab. The incremental costs per QALY were higher for nivolumab (154,869$) and lower for pembrolizumab (133,672$) (Table 7).

### Perspectives

After a long period of small improvements in the treatment of NSCLC (introduction of 3rd generation drugs, concomitant chemo-radiotherapy in stage III, adjuvant chemotherapy for selected surgical stages), a first revolution occurred with targeted therapies. Unfortunately, those very active drugs concerned a limited fraction of metastatic NSCLC. Discovery of checkpoint inhibitors, mainly antiPD1 and antiPDL1 antibodies profoundly modified in a few years the algorithm strategy for advanced NSCLC. Many questions are unresolved. Despite clinical effectiveness in most of the patients, a significant number had a rapid progression, up to 30% at first tumor assessment. On the opposite, 10–15% of the patient had a very long-term disease control. Currently, we do not have sensitive and specific marker for individual prediction of immunotherapy effectiveness.

The activity of antiPD1/PDL1 was first demonstrated in unresectable, non-irradiable NSCLC. The first demonstration of adjuvant durvalumab (43, 44) in unresectable stage III NSCLC resulted in major improvement in PFS and OS after concomitant chemo-radiotherapy. Further trials are in progress as well after concomitant or sequential chemo-radiotherapy. Introduction of immunotherapy in resectable stage I-III NSCLC showed major pathological responses, despite sometimes a poor clinical response rate after a short period of treatment (25–28). Other trials are on the way for induction before surgery [Atezolizumab in Stage IB, II, or IIIA (NCT02927301), Durvalumab (NCT03030131), Nivolumab in Stage IB-IIIA (NCT02595944), Pembrolizumab in N2 disease (NCT03053856)]. But the largest randomized trials are now conducted in adjuvant setting in stage IB-IIIA completely resected NSCLC (PEARLS: “Adjuvant Pembrolizumab for resected NSCLC” and BR31 NCI-IFCT trial: “Adjuvant durvalumab in resected NSCLC”). However, the neoadjuvant setting might be more promising as the tumor is still in place, which might prime the immune system more efficiently.

Based on the immune cell control (84), we may expect improve efficacy by combining agents working at different levels of the system, which is yet done with antiPD1/PDL1 and antiCTLA4. Chemotherapy delivering tumor antigen is able boosting the immune system in addition to immunotherapy. Other options that are in development include e.g., co-administration of radiotherapy (85), targeted agents or vaccines. New antiPD1 and antiPDL1 are also under development.

Besides PDL1 and CTLA4, other potential therapeutic targets were discovered and clinical trials are underway. Immune regulators outside of the PD-1/PD-L1 axis are e.g., myeloid-derived suppressor cells (MDSCs), tumor-associated macrophages (TAMs), NK cells, dendritic cells, B-cells… Three main scenarios for further investigations are release of cancer antigens (vaccination, adoptive cell therapy), activation of the T-cell response (TNF-R superfamily), and regulation of the inhibitory immune response (Ig superfamily, metabolites and myeloid cell factors) (86) with a lot of interesting new drugs (87). Among them, we may cite IDO inhibitors as epacadostat, although the first results were disappointing.

Other areas for future investigation are the optimal duration of ICI therapy (fixed duration or until progression/unacceptable toxicity), and the optimal dose and cycle duration. In stage III for example, 1 year of durvalumab resulted in a superior survival compared to placebo (44). However, there are no available trial results suggesting that a longer duration of adjuvant durvalumab would be more beneficial, or that a shorter duration would cause no harm to the patient. In contrast, in stage IV NSCLC the results of the CheckMate153 trial suggest that discontinuing treatment after 1 year results in a shorter survival compared to continuing nivolumab (88). A fixed duration of 2 years of treatment vs. continuing ICI resulted in a similar 3-year survival in nivolumab and pembrolizumab treated patients, but number of patients reaching the 2 year of treatment was low (15, 31, 89). It is currently unclear whether re-challenge ICI in patients relapsing after ICI discontinuation is a good treatment strategy as results for pembrolizumab (79% of rechallenged patients had clinical benefit) and nivolumab (59% clinical benefit) are somewhat conflicting (88, 89). Type of response is associated with outcome (responders have a better outcome than those with stable disease) (66), but in contrast to melanoma (suggested that in those with a complete response ICI can be stopped) (90), it is in NSCLC not clear whether type of response can be used in the decision whether to discontinue the ICI. It would be interesting to prospectively evaluate whether complete metabolic PET response and/or complete ctDNA clearance could aid in the decision to continue or to stop the ICI.

Fixed dosing of ICI is common now, but it is resulting in financial toxicity in both the USA and in Europe (91). Nivolumab fixed dose (240 mg/2 weeks or 480 mg/4 weeks) corresponds to a body weight of 80 kg, pembrolizumab 200 mg/3 weeks corresponds to a body weight of 100 kg. However, the mean weight in the USA is 82 kg, is 72 kg in Europe (92, 93) and is probably lower in cancer patients. Research should focus on whether ICI dose can be reduced (in all patients or in responders), or whether duration of a cycle can be lengthened, as this would cause a reduction in cost and a longer cycle duration would result in more comfort for the patient. Rationale for lengthening of a cycle is that the half-life of antiPD-(L)1 is between 12 and 90 days regardless of the dose, and that occupancy of PD-1 on T-cells lasts ~3 months (12).

## Conclusions

Based on high-level evidences (randomized trials and meta-analyses), immunotherapy profoundly modified our therapeutic algorithms in advanced/metastatic NSCLC. From quite disappointing long-term results of conventional platinum-based chemotherapy, we are now moving to amazing long-term survival rates, which can grow up to 10% 5-year rates in heavily pre-treated metastatic NSCLC.

A lot of questions should be resolved in order allowing a sustained economic approach of these expensive drugs as the best duration of treatment, the adequate choice of those patients who will most benefit, the optimal combination…

AntiPD1 and antiPDL1 opened a new window for treating NSCLC. We have now to determine the best ways using these very active anticancer agents.

## Author Contributions

TB and VD performed the systematic review (search equation, abstract selection, and data extraction). TB, A-MD, and LH reviewed and adapted the manuscript. All authors agreed on the content of the manuscript.

### Conflict of Interest

A-MD attended advisory boards and/or provided lectures for: Roche, Eli Lilly, Boehringer Ingelheim, Astra Zeneca, Pfizer, BMS, Amgen, Novartis, MSD, Takeda, Clovis; for which her institute received honoraria. AD received research support from BMS and AbbVie. LH reports Grants/research support from Roche, Boehringer, AstraZeneca (institution), personal fees from Quadia, BMS, Roche, Boehringer Ingelheim. TB was investigator for clinical trials (Astra Zeneca, Pfizer, Novartis, Peregrine, MSD). The author declares that the research was conducted in the absence of any commercial or financial relationships that could be construed as a potential conflict of interest.
