# Supplementary material for: Immunotherapy: From Advanced NSCLC to Early Stages, an Evolving Concept
Source: Front Med (Lausanne). 2020 Mar 24;7:90. doi: 10.3389/fmed.2020.00090 (PMC7105823; doi:10.3389/fmed.2020.00090)
Supplement: Supplementary file 1 [file Data_Sheet_1.docx]

Appendix 1. Search strategy Medline

List of MeSH terms and free-text keywords used to search Ovid Medline database

Database: Epub Ahead of Print, In-Process & Other Non-Indexed Citations, Ovid Medline® Daily and Ovid Medline® 1946-present

| **P criterion** | **Searched MeSH terms, free-text keywords and phrases** |
| --- | --- |
| Lung Cancer | P = lung neoplasms/ or bronchial neoplasms/ or carcinoma, bronchogenic/ or carcinoma, non-small-cell lung/ or small cell lung carcinoma/ or pancoast syndrome/ or pulmonary blastoma/ or lung neoplasm*.ti,ab. or lung carcinoma*.ti,ab. or lung tumour*.ti,ab. or lung tumor*.ti,ab. or pulmonary neoplasm*.ti,ab. or pulmonary carcinoma*.ti,ab. or pulmonary tumour*.ti,ab. or pulmonary tumor*.ti,ab. or bronchial neoplasm*.ti,ab. or bronchial cancer*.ti,ab. or bronchial carcinoma*.ti,ab. or bronchial tumour*.ti,ab. or bronchial tumor*.ti,ab. or bronchogenic neoplasm*.ti,ab. or bronchogenic cancer*.ti,ab. or bronchogenic carcinoma*.ti,ab. or bronchogenic tumour*.ti,ab. or bronchogenic tumor*.ti,ab. or pancoast* syndrome*.ti,ab. or pancoast* tumor*.ti,ab. or pancoast* tumour*.ti,ab. or ((lung.ti,ab or pulmonary.ti,ab) and (cancer*.ti,ab OR neoplasms/)) |
| **I criterion** | **Searched MeSH terms, free-text keywords and phrases** |
| Definition of immunotherapy | (exp Immunotherapy/ OR immunotherap*.ti,ab OR immunization*.ti,ab OR vaccin*.ti,ab OR Nivolumab.ti,ab,nm OR Opdivo.ti,ab,nm OR Pembrolizumab.ti,ab,nm OR Keytruda.ti,ab,nm OR Atezolizumab.ti,ab,nm OR MPDL3280A.ti,ab,nm OR Durvalumab.ti,ab,nm OR Avelumab.ti,ab,nm OR Ipilimumab/ OR ipilimumab.ti,ab,nm OR yervoy.ti,ab,nm OR tremelimumab.ti,ab,nm OR ticilimumab.ti,ab,nm) |
| **Selection criteria** | **Searched MeSH terms, free-text keywords and phrases** |
| Type of study | (clinical trial phase iii.pt or clinical trial phase iv.pt or comparative study.pt or controlled clinical trial.pt or guideline.pt or meta analysis.pt or practice guideline.pt or randomized controlled trial.pt or systematic review.pt or clinical trial phase iii.ti,ab or clinical trial phase iv.ti,ab or comparative study.ti,ab or controlled clinical trial.ti,ab or guideline*.ti,ab or meta analysis.ti,ab or randomized controlled trial.ti,ab or systematic review.ti,ab or population based.ti,ab or systemic review.ti,ab or systematic overview.ti,ab or randomised trial.ti,ab. or randomized controlled study.ti,ab) |

Legend : term/ = MeSH term (with all the possible subheading combinations)

exp = exploted MeSH term, meaning that this MeSH term and all the MeSH terms found below in the hierarchy are taken into consideration.

Appendix 2. Flow chart of studies’ selection

Identification

Records excluded, duplicates

(n=0)

Relevant records identified through database searching

(n= 966)

Eligibility

Screening

Included

Studies included in systematic review

(n= 70)

Articles identified through other sources

(n= 16) (including abstracts)

Studies met inclusion-exclusion criteria

(n= 51)

Full-text articles retrieved for more detailed evaluation

(n= 86)

Records excluded based on title/abstract

(n= 880)

Records screened based on title/abstract

(n= 966)

Articles excluded based on inclusion-exclusion criteria

(n= 19)

Articles not considered because updated data (n= 16)
